# Gender Influence on XOR Activities and Related Pathologies: A Narrative Review

**DOI:** 10.3390/antiox13020211

**Published:** 2024-02-07

**Authors:** Andrea Bolognesi, Massimo Bortolotti, Maria Giulia Battelli, Letizia Polito

**Affiliations:** Department of Medical and Surgical Sciences—DIMEC, Alma Mater Studiorum, University of Bologna, Via San Giacomo 14, 40126 Bologna, Italy; massimo.bortolotti2@unibo.it (M.B.); letizia.polito@unibo.it (L.P.)

**Keywords:** cardiovascular disease, chronic kidney disease, gender medicine, gout, hypertension, metabolic syndrome, uric acid, xanthine oxidoreductase

## Abstract

Taking into account the patient’s gender is the first step towards more precise and egalitarian medicine. The gender-related divergences observed in purine catabolism and their pathological consequences are good examples of gender medicine differences. Uric acid is produced by the activity of xanthine oxidoreductase (XOR). The serum levels of both XOR activity and uric acid differ physiologically between the genders, being higher in men than in women. Their higher levels have been associated with gout and hypertension, as well as with vascular, cardiac, renal, and metabolic diseases. The present review analyzes the gender-related differences in these pathological conditions in relation to increases in the serum levels of XOR and/or uric acid and the opportunity for gender-driven pharmacological treatment.

## 1. Introduction

The profound gender differences in the fields of physiology and pathology, deriving from Hippocratic medicine, seem to have been overcome in contemporary medicine [1]. However, despite the goal of gender equality, a 70 kg Caucasian male has often been taken as the unique representative model for standard medicine and a therapeutic set point [2]. This approach has been proven in many cases to be almost as wrong as the Hippocratic one [3]. Our contemporary biomedical knowledge is often still steeped in ancient myths and prejudices. New voices are now rising up, demanding a more modern view of gender medicine, which is the first and essential step towards a new and truly gender-equitable approach to therapy.

The gender-related divergences observed in purine catabolism and their pathological consequences are good examples of gender medicine differences. It is known that uricemia is higher in men than in women, with normal range values of 3.4–7.0 mg/dL for men versus 2.4–5.7 mg/dL for women [4]. At least in part, this difference is due to the uricosuric effect of estrogens, which reduce the risk of hyperuricemia in premenopausal women. The level of uricemia increases with age from childhood to senescence with a similar trend in the genders until puberty, whereupon the curve slopes diverge [5]. While in men, the values continue to grow, in women, the level of uricemia shows a plateau during their fertility period and starts growing again after menopause [6].

**Figure 1:**
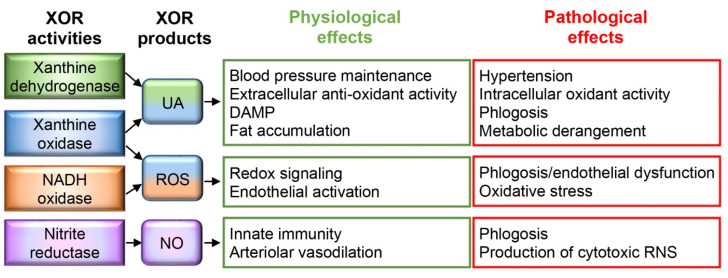
Xanthine oxidoreductase (XOR) activities and products: physiological and pathological effects. Uric acid (UA), produced by the xanthine dehydrogenase and oxidase activities of XOR, has several physiological effects: (i) regulation of blood pressure by activating the renin–angiotensin–aldosterone system, (ii) antioxidant activity, which is fundamental in biological fluids, (iii) macrophage stimulation by acting as a damage-associated molecular pattern (DAMP), (iv) fat accumulation by regulating lipidic and glycidic metabolism. However, hyperuricemia can have pathological effects, promoting hypertension and chronic inflammation, as well as inducing hypertriglyceridemia and insulin resistance. In addition, intracellular UA accumulation and UA oxidant products contribute to oxidative stress. Superoxide ion and hydrogen peroxide (ROS), produced by xanthine oxidase and reduced nicotinamide adenine dinucleotide (NADH) oxidase activities, have a redox signaling function that is essential for innate immunity because it is implicated in bactericidal action as well as cell activation, proliferation, and migration. On the other hand, excess amounts of ROS can induce endothelial dysfunction and oxidative stress. Nitric oxide (NO), produced by the nitrite reductase activity of XOR, promotes local arterial vasodilation and contributes to innate immunity by generating reactive nitrogen species (RNS). High levels of NO can induce phlogosis and tissue damage by producing cytotoxic RNS, such as peroxynitrite ion [16].

Uric acid is produced by xanthine oxidoreductase (XOR), which is a NAD^+^-dependent dehydrogenase (XDH, EC 1.1.1.204) in the cell, but when released in biological fluids, it can be converted into an oxidase (XO, EC 1.1.3.22) as a consequence of the oxidation of a couple of specific thiol groups or their loss following partial proteolysis [7,8]. In acidic and hypoxic conditions, XOR can have NADH oxidase activity, also producing reactive oxygen species (ROS), as well as nitrite reductase activity, which generates nitric oxide (NO) (Figure 1) [9].

The level of XOR activity in human serum is very low (0–0.5 mU/L) in normal controls, reaching up to 1.8 mU/L in patients with apparently no evidence of hepatic or intestinal pathologies [10]. Indeed, most of the serum XOR is released from the liver [11,12]. XOR activity is determined by monitoring the formation of urate through absorbance at 295 nm [11], or by using ^14^C-xanthine as a substrate and measuring the uric acid produced after separation with high-performance liquid chromatography [13]. A high level of XOR activity is only present in the liver and small intestine, starting from the fetal period [13], as well as in the breast during lactation [13,14]. The XOR activity in liver samples from patients with normal hepatic function was 20% higher in men than in women [15], suggesting that another reason for the observed difference in the level of uricemia between the genders could depend on the liver dimension and the consequent amount of available XOR.

In this narrative review, we examine the gender-related differences in the pathological increase in XOR and/or uric acid serum levels in order to evaluate whether a pharmacological treatment should be differently recommended on the basis of the patient’s gender. The bibliography was searched in the PubMed and Google Scholar databases, using the keywords “cardiovascular disease”, “chronic kidney disease”, gender”, “gout”, “hypertension”, “metabolic syndrome”, “uric acid”, “uricemia”, and “xanthine oxidoreductase” in appropriate combinations.

## 2. Gout

Hyperuricemia is a well-known risk factor for gout, which is the most widespread type of inflammatory arthritis. Long-term studies on asymptomatic hyperuricemic patients (serum urate concentration ≥9 mg/dL) showed that only 22% of them developed gouty arthritis after 5 years [17], and about 30% of them developed clinically evident gout over 15 years [18]. For this reason, the American College of Rheumatology’s guidelines for the management of gout recommend not starting a urate-lowering therapy (ULT) for asymptomatic hyperuricemia, also in consideration of the potential negative effects of this therapy [19].

The augmentation of circulating uric acid can be the consequence of genetic factors, the excessive intake of fructose, purine-rich food, or alcohol, or of tumor lysis syndrome, as well as of a pathological decrease in renal or intestinal uric acid excretion [20,21,22].

A prospective cohort study, which examined 2476 women and 1951 men in Massachusetts for a period of 52 years, showed that the risk of developing gout is higher for men than for women with the same level of uricemia [23]. In addition, this risk has been associated with growing age, obesity, alcohol consumption, hypertension, and diuretic use. Such an association highlights the relation between hyperuricemia and cardiometabolic and kidney diseases [23,24]. In a population study enrolling 5525 Taiwanese subjects, including 1568 (28% women) asymptomatic hyperuricemic patients and 347 (18% women) gout patients, hyperuricemia was significantly associated with unfavorable left ventricular remodeling and tightly linked to diastolic dysfunction. Despite similar degrees of left ventricular remodeling, hyperuricemic and gouty women showed worse diastolic indices than men, suggesting the need for more stringent follow up for women [25].

However, a retrospective cohort study that included 1565 gout patients from the Netherlands reported no gender differences in the response to ULT with the XOR inhibitor allopurinol (255 women and 1045 men) or the uricosuric drug benzbromarone (60 women and 205 men) after correction for confounding factors [26].

## 3. Hypertension

**Figure 2:**
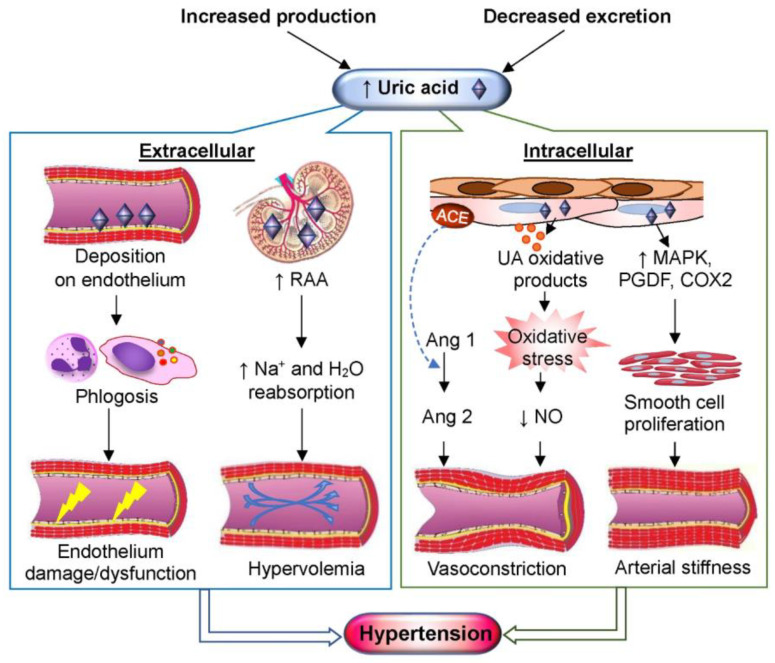
Uric acid (UA) and hypertension. The augmentation of circulating UA can be the consequence of genetic factors, excessive intake of fructose, purine-rich food or alcohol, or tumor lysis syndrome, as well as of a pathological decrease in renal or intestinal UA excretion. An excess amount of UA can lead to hypertension through different mechanisms. Extracellularly, the deposition of urate crystals in main vessels may trigger a proinflammatory response, thus causing direct endothelial injury and dysfunction [35]. UA can activate the renin–angiotensin–aldosterone (RAA) system by increasing the juxtaglomerular renin release. Thus, the reabsorption of sodium (Na^+^) and water (H_2_O) is augmented in distal tubule, thus inducing hypervolemia. The renin release augments the formation of angiotensin 1 (Ang 1), which is converted into angiotensin 2 (Ang 2) by the endothelial angiotensin-converting enzyme (ACE). Intracellularly, UA oxidative products can cause oxidative stress and induce vasoconstriction by lowering the availability of endothelial nitric oxide (NO). Moreover, intracellular accumulation of UA induces the proliferation of smooth muscle cells, which leads to arterial stiffness by increasing the activity of mitogen-activated protein kinases (MAPK), platelet-derived growth factor (PDGF), and cyclooxygenase 2 (COX-2) [36].

Hyperuricemia negatively influences endothelial function, lowering the availability of NO and thus contributing to trigger the initial phases of hypertension (Figure 2), which is in turn involved in cardiovascular (CVD) [27] and chronic kidney (CKD) [28] diseases. The gender differences in hypertension are influenced by the effects of estrogens, which increase the NO availability in the arteriolar endothelium [29] via both genomic and nongenomic mechanisms [30].

The blood pressure control rate and cardiovascular risk profile (BP-CARE) study included 3206 hypertensive patients (49.6% women) recruited from central and eastern European countries and analyzed gender-related differences in the correlation between hyperuricemia and cardio-nephro-metabolic variables. Hyperuricemia was statistically associated with diabetes mellitus, CVD, and CKD in both genders, but its correlation with metabolic syndrome and blood pressure was significant only in men [31].

A cross-sectional study including 1776 Chinese adults aged 45–60 years (41% women) was conducted to evaluate the gender difference in the relationship between hyperuricemia and hypertension; the association was significantly higher in men than in women. Similarly, the fasting glycemia level, body mass index, and waist circumference were significantly higher in men than in women [32].

The association between plasma XOR activity and hypertension was investigated in 271 subjects (119 men and 152 women) taking part in the Tanno–Sobetsu Study. The subjects were nondiabetic and had not taken any medications. The plasma XOR activity was measured using a combination of liquid chromatography and triple quadrupole mass spectrometry to detect [^13^C_2_, ^15^N_2_]-uric acid, using [^13^C_2_, ^15^N_2_]-xanthine as a substrate. The plasma XOR activity was higher in men than in women and significantly higher in hypertensive patients than in the group without hypertension, although no difference was observed in the level of uricemia between the two groups. After adjusting for confounding parameters, a high level of XOR activity in the plasma was an independent risk factor for hypertension. However, after dividing the subjects by gender, the differences observed in the plasma XOR activity between the patients with and without hypertension were significant only in men [33].

In some cases, contrasting results have also been observed with previous ones. A Japanese adult population that included 14603 men and 8391 women who received annual health examinations was followed for 10 years to evaluate whether hyperuricemia is a risk factor for hypertension. A significant association was reported between the basal level of uricemia and an increase in systolic blood pressure over time in women but not in men [34].

In conclusion, the results of the above-reported studies, conducted on different populations, prompt the need for more in-depth gender-specific control of the serum uric acid level in relation to hypertension.

## 4. Vascular Diseases

The atherosclerotic process is both the cause and the consequence of hypertension, which contributes to arterial changes together with hyperglycemia, diabetes mellitus, dyslipidemia, and smoking.

In a 3-year study that included 648 Japanese adults (40% women) undergoing antihypertensive treatment, their flow-mediated vasodilatation, brachial–ankle pulse wave velocity, and common carotid artery intima–media thickness were measured to evaluate their association with the level of uricemia. The results showed a cross-sectional and longitudinal association between uricemia and the above-cited vascular markers in women but not in men [37].

The difference between systolic and diastolic blood pressure is called the pulse pressure and is a predictor of arterial stiffness, which is a risk factor for CVD [38] and CKD [39]. The analysis of data from the seventh Korean National Health and Nutrition Examination Survey, including 2800 men and 3510 women, indicates that both the systolic and pulse pressures are positively associated with the level of uricemia in women, but not in men [40].

The correlation of the serum level of uric acid with the ambulatory arterial stiffness index, which is used to assess vascular function, was significantly negative in men but positive in women after adjusting for confounders, in a study including 226 male and 140 female patients from Spain [41]. In addition, a cross-sectional study including 207 women and 131 men from Brazil showed that the uricemia level was significantly associated with the presence of arteriolopathy, as measured by the internal carotid resistive index in hypertensive women but not in men [42].

The association between the uricemia level and the low ankle–brachial index, as a marker of peripheral arterial disease (PAD), was investigated in 6262 Chinese patients with high CVD risk. After adjusting for confounding cardiometabolic parameters, the association was significant only in women, suggesting that in this gender, hyperuricemia is associated with a higher risk of PAD [43].

A recent review evidenced that patients with hyperuricemia are at high risk for developing PAD [44]. However, considering patient gender, controversial results were obtained. A study enrolling patients with hypertension and PAD (about 75% being men) evidenced that the presence of hyperuricemia was associated with more pronounced claudication in a treadmill test [45]. In another study, serum uric acid was found to be an independent risk factor for PAD in women, but not men, with diabetes [46]. However, this gender difference was reversed in a study enrolling patients with asymptomatic hyperuricemia, in which serum uric acid had a significant independent association with PAD in men but not in women [47].

Vascular pathologies are strongly influenced by the conditions of the endothelium. Endothelial dysfunction can be induced by an increase in ROS produced by XOR activity, or it can depend on the action of hormones. The ability of estrogens to promote the release of NO and prevent the production of ROS, and the consequent chronic inflammation, caused by uric acid metabolism, is well known. Furthermore, estrogens regulate the level of uric acid by inducing the expression of transmembrane urate transporters [48]. In addition, estrogens can regulate renal urate transporter expression and localization directly or by activating specific transcription factors [21].

A retrospective cross-sectional analysis of 140 (61.4% women) adult US patients investigated the relationship between the level of uricemia and the peripheral endothelial dysfunction evaluated by reactive hyperemia peripheral arterial tonometry. In a multivariate analysis, after adjusting for covariables, elevated serum uric acid levels, although in the normal range, were significantly associated with peripheral endothelial dysfunction only in women, who were otherwise at low risk for CVD [49].

The prevalence of coronary artery disease was significantly higher in men than in women in the Novara Atherosclerosis Study Group (NAS), which recruited 3520 Italian patients (69% men, 31% women). Men showed a significantly higher level of serum uric acid, as expected. However, a significant relationship between uricemia and coronary artery disease was observed only in women [50].

A new measure of arterial stiffness is represented by the cardio-ankle vascular index, which reflects the stiffness from the ascending aorta to the ankle arteries, independently of blood pressure, and may be clinically useful for assessing the risk of CVD. A retrospective study was conducted with 1217 Chinese subjects (696 men and 521 women) who underwent a routine health examination. The percentage of hyperuricemic women with a high cardio-ankle vascular index was three times that of women without hyperuricemia and of men with or without hyperuricemia [51].

The plasma XOR activity was determined in 132 Japanese patients (59% men, aged 62 ± 13 years; 41% women, aged 68 ± 8 years) suspected of having coronary artery spasm and, for this reason, were subjected to an intracoronary acetylcholine provocation test. As expected, both the uricemia and XOR activity were higher in the men than in the women. A high level of plasma XOR activity was significantly predictive of coronary artery spasm in both men and women, but the incidence of such spasm was significatively higher in women, and the threshold value of XOR activity for predicting the incidence of coronary artery spasm was significantly lower in women than in men. Considering the age of the women patients, these results suggest that the loss of estrogen protection has a role in coronary artery pathology and that XOR-derived ROS could contribute by decreasing the NO availability [52].

However, some contrasting results have also been reported.

A study recruited 3686 patients (48% men) aged 54 to 79 years, from 5 European countries, with at least 3 CVD risk factors but free of any previous cardio-or cerebrovascular event at baseline. The patients were followed for about 3 years, and vascular events were recorded concerning the brain, heart, or a lower extremity. A U-shaped association between the uricemia level and vascular events, in particular, the progression of the carotid intima–media thickness, was observed with a significant increase in CVD risk in men but not in women [53].

Considering the uricosuric effects of estrogens, the level of circulating uric acid is indicative of the production by XOR in women. The greatest prevalence of the adverse vascular effects of hyperuricemia in women suggests that the increase in uricemia is due to increased XOR activity, and both have a primary role in inducing the consequent oxidative stress, which is in turn responsible for vascular changes.

## 5. Cardiac Diseases

Although CVD is the main cause of death among women, this gender is protected during the reproductive period compared to age-matched men, while the incidence of CVD increases post-menopause. The mechanisms through which estrogens reduce CVD incidence, or at least delay their occurrence, have been discussed elsewhere and include the reduction of mitochondrial ROS production and the greater expression of both endothelial NO synthase and vascular endothelial growth factor. In addition, estrogens activate some antioxidant mechanisms and reduce both the activation of fibrocytes and the deposition of collagen. Finally, estrogens have a downregulating effect on the angiotensin-converting enzyme [54].

The Losartan Intervention For Endpoint Reduction in Hypertension (LIFE) study enrolled 9173 (46% men, 54% women) US and Scandinavian patients aged 55 to 80 years with hypertension and electrocardiographic signs of left ventricular hypertrophy. The results of this investigation ascertained an association between the level of uricemia and the overall occurrence of cardiovascular events, such as myocardial infarction, stroke, and cardiovascular death. However, when analyzed by the gender, the association was significant in women but not in men [55]. Consistently, a positive correlation was observed between an increase in the level of serum uric acid and the occurrence of heart failure and cardiac death only in women among 1380 Japanese patients (76% men, 24% women) aged 53 to 83 years with acute coronary syndrome, who were enrolled in the Ibaraki Cardiac Assessment Study [56].

The correlation between the level of uricemia and cardiac diastolic dysfunction was investigated in 744 Japanese cardiac patients (73% men, 27% women) aged 56 to 82, with preserved left ventricular ejection fraction. The prevalence of diastolic dysfunction increased in women, but not in men, with a relatively higher serum uric acid level, even within the normal range [57].

Among 161 (62% men, 38% women) Chinese adult patients with obstructive hypertrophic cardiomyopathy, the level of uricemia was significantly associated with left ventricular mass index in women but not in men, after adjusting for a potential confounding factors [58].

The level of uricemia was significantly associated with a high 10-year arteriosclerotic CVD risk for women but not for men in a study including 2537 type 2 diabetic Chinese patients (1068 men, 1469 women) aged 64–75 years [59].

The relationship between the level of uricemia and the risk of CVD was studied in adult Chinese people belonging to the following ethnic groups: Dong (5978 subjects, 34% men), Miao (4790 subjects, 36% men), and Bouyei (5199 subjects, 30% men). After adjustment for potential risk factors, hyperuricemia was positively associated with the risk of CVD and the risk of stroke in older women of the ethnic groups of Bouyei and Dong, respectively. However, the multivariate logistic regression analysis did not show any association in the women of the Miao ethnic group, nor in the men of any of the three ethnic groups [60].

The prevalence of cardiometabolic and kidney disease with different levels of uricemia was studied in a German adult population including 6918 participants (51% women). A significantly higher association of uricemic values >5 up to 6.8 mg/dL with hypertension and diabetes was observed in women compared to men. A significant association was also reported between hyperuricemia and coronary heart disease, heart insufficiency, and chronic kidney insufficiency in women [61].

The reported studies suggest that routine measurement of the serum uric acid level is indicated for older female patients with CVD and pose the question of when urate-lowering therapy (ULT) could be recommended in these patients. Furthermore, the differences observed among the women of different ethnic groups highlight the need to take into account the genetic specificity of populations, as well as the different cultures, which determine significant differences in nutritional habits and lifestyle.

## 6. Kidney Diseases

Hyperuricemia can affect the progression of kidney failure by promoting hypertension, chronic inflammation, and oxidative stress.

The association between hyperuricemia and indicators of CKD was investigated in a cross-sectional study of 7053 Chinese adults (67% women). As expected, the level of uricemia and the prevalence of hyperuricemia were significantly higher in men than in women. Hyperuricemia was independently associated with an increased risk of CKD, and this association was significantly stronger in men than in women [62].

In a cross-sectional study that recruited 6776 US women aged 24–63 years without hypertension or diabetes, each 1 mg/dL increase in uricemia was associated with a 39% increased prevalence of CKD, but only for a serum uric acid level >4.5 mg/dL, a threshold under which there was no association. This association was stronger in older women [63].

A retrospective analysis was performed on 578 young adult Korean patients (86% women) with lupus nephritis who underwent kidney biopsy to determine the association of hyperuricemia with long-term output after 7 ± 4 years. Hyperuricemia was associated with a significantly higher progression of kidney damage. This association was still positive in women but not in men after adjusting for confounding covariates [64].

A large cohort, consisting of 26,645 adult Taiwanese subjects (9356 men, 17289 women) without CKD at baseline, was analyzed in a longitudinal study with follow-up examinations after about 4 years to explore any gender differences in the relationships between hyperuricemia and the decline of renal function. Hyperuricemia was significantly associated with a low estimated glomerular filtration rate (eGFR) in both men and women but was more strongly associated with new-onset CKD in women compared to men [65].

Hyperuricemia is strongly linked to altered renal function both as a causal mechanism and as a consequence since the altered renal function determines a compromised clearance of uric acid from the blood, and consequently, hyperuricemia.

## 7. Metabolic Diseases

Hyperuricemia alters both glycidic and lipidic metabolisms by favoring insulin resistance, dyslipidemia, and fat accumulation. Indeed, XOR-derived reactive oxygen species and uric acid could contribute to the pathogenesis of metabolic syndrome by promoting hypertension, insulin resistance, obesity, and hypertriglyceridemia [66].

The level of plasma XOR activity was significantly associated with diabetes mellitus, dyslipidemia, and hyperuricemia as well as with body mass index, waist circumference, insulin resistance, eGFR, and the plasma level of hepatic enzymes, triglycerides, fasting glucose, insulin, and glycated hemoglobin in the Tanno–Sobetsu Study involving 627 Japanese subjects (292 men, 335 women) aged 65 ± 15 years [67].

In a Chinese population enrolling 17,762 subjects (10,912 men, 6850 women), a stronger association between the serum uric acid level and metabolic syndrome markers was reported in women compared to men, suggesting that the same level of uricemia may correspond to uneven effects between genders on lipid metabolism and blood pressure. Before menopause, young women with hyperuricemia had the highest risk of developing metabolic syndrome. However, the prevalence of metabolic syndrome was higher in men than in women before 60 years, with the opposite occurring afterwards, underlining the relevance of the role of sex hormones in the occurrence of metabolic alterations [68].

Another study enrolling 1006 Chinese adults (617 men, 759 women, aged 45 to 59 years) analyzed gender differences in the association between uricemia level and nonalcoholic fatty liver disease. After adjustment by age, diastolic blood pressure, fasting glucose, triglycerides, total cholesterol, high- and low-density lipoprotein cholesterol, and obesity, the association, although significant in both genders, was stronger in men than in women [69].

A total of 20,207 diabetic Chinese patients (8541 men, 11,666 women) aged 64–78 years were investigated to assess gender differences in the association between hyperuricemia and diabetic kidney disease. Hyperuricemia was significantly associated with albuminuria and decreased eGFR in both genders; however, a stronger association was observed in men than in women, after adjustment for confounding factors [70].

A cross-sectional examination was performed in a military cohort of 7504 (6738 men, 766 women) young adult Taiwanese subjects to assess the gender-specific association of hyperuricemia with metabolic abnormalities. Hyperuricemia was associated with high blood pressure, low high-density lipoprotein, high triglycerides, high low-density lipoproteins, high fasting plasma glucose, and central obesity in both men and women, but the association of these metabolic syndrome markers with hyperuricemia was significant only in men [71].

The Beijing Health Management Cohort study enrolled 8237 adult Chinese subjects (5471 men, 2766 women) who were free of diabetes, prediabetes, CVD, and cancer at baseline in a 6-year longitudinal investigation. The purpose of the study was to verify the association between uricemia and prediabetes, defined as a fasting plasma glucose level of 6.0–6.9 mmol/L, where 5.55 mmol/L was considered the limit of normality. The association between prediabetes and the level of uricemia was significant in women aged 48 years or older, while no association was detected in men, suggesting a connection with the hormonal trend [72].

A total of 325 subjects (234 men, 91 women) aged 31–46 years were enrolled in a study to determine the association between the serum levels of XOR protein and type 2 diabetes in a Bangladeshi adult cohort. The XOR level, measured by enzyme-linked immunosorbent assay, was significantly higher in women than in men, as well as in diabetic and pre-diabetic patients compared to control subjects. On the contrary, the level of uricemia was significantly higher in men than in women, as well as in control subjects compared to pre-diabetic and diabetic patients. The serum fasting glucose was directly proportional to the XOR level and inversely to the uricemia level, suggesting that the XOR serum level can be a better marker than uricemia in patients with type 2 diabetes [73].

In an animal model, represented by Goto-Kakizaki rats, aging was demonstrated to induce the development of type 2 diabetes. Female diabetic rats showed greater glucose uptake by the brain, cerebellum, and heart and a higher insulin sensitivity in a glucose tolerance test, as well as a lower production of free radicals than male diabetic rats. Furthermore, estrogen replacement therapy was able to improve the insulin sensitivity and prevent the increase in oxidative stress in diabetic ovariectomized females. In addition, the plasma level of XOR activity was higher in male diabetic rats compared to female, underlining the role of both hormones and oxidative stress in the development of diabetes [74].

Liver-specific *Xdh* gene deletion in mice demonstrated that hepatocyte XOR activity is necessary for the development of hyperuricemia. However, reduction of hepatic and plasmatic uric acid failed to ameliorate the metabolic alterations of obesity, suggesting the role of hyperuricemia as a biomarker of obesity, without any involvement in the pathogenesis [75].

Female mice, in which obesity was induced by diet, showed alterations in their glucose and lipid metabolism and hepatic inflammation. In addition, increased expressions of superoxide dismutase, heme oxygenase, and xanthine dehydrogenase were observed. However, these animals did not show hyperuricemia, probably because of the uricosuric effect of estrogens [76]. These somewhat countervailing results support the conclusion that much work is yet to be accomplished before assigning a pathogenetic role to the observed correlations.

In gender differences related to metabolic diseases, the role of age, and consequently, of the level of estrogen appears to be decisive.

## 8. Conclusions and Perspectives

In this review, we have discussed gender-related differences in the pathological increase in XOR activity and/or uric acid serum levels. The main results discussed above are summarized in Table 1.

XOR activity can produce uric acid, as well as reactive oxygen and nitrogen species, which all have physiological functions when generated at the proper level, but at higher levels, they may contribute to oxidative stress and promote various disorders.

Although uric acid is an XOR product, the level of uricemia is not related to XOR plasma activity because it derives mainly from liver metabolism and also depends on kidney and intestinal excretions. In turn, hyperuricemia influences the functioning of the kidney and cardiovascular system and directs the glycidic and lipid metabolisms, thus representing both a marker and a causal agent of illness. This also explains why hyperuricemia is present in pathological situations concerning different organs and systems.

**Figure 3:**
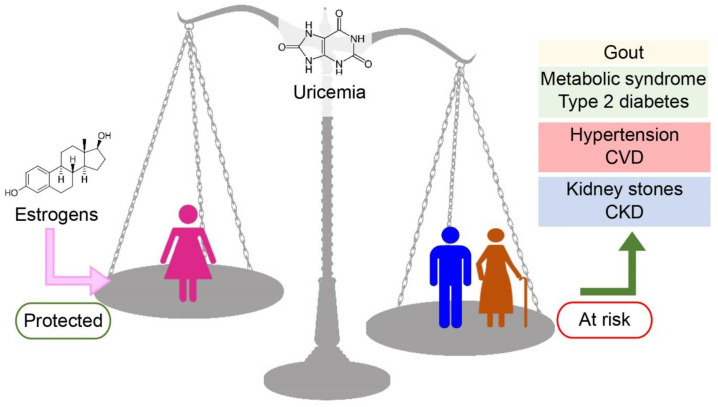
Correlations among uricemia, gender, and age, and metabolic, cardiovascular, and renal diseases. Uricemia is lower in women than in men, with normal range values of 3.4–7.0 mg/dL for men versus 2.4–5.7 mg/dL for women. Such a difference is due, at least in part, to the uricosuric effect of estrogens, which reduce the risk of hyperuricemia in premenopausal women, thus protecting young women from hyperuricemia-related diseases. While in men, the values of uricemia slightly grow for their whole lives, in women, the level of uricemia shows a plateau during their fertility period and starts growing again after menopause. The levels of uricemia are correlated to the onset of several diseases. Hyperuricemia can cause gout and can contribute to the development of metabolic syndrome and type 2 diabetes as well as hypertension, cardiovascular disease (CVD), urate kidney stones, and chronic kidney diseases (CKD).

Hyperuricemia occurs more often in men than in women, and consequently, gout affects men more than women (Figure 3).

In addition, increasing levels of uricemia and XOR plasma activity have been correlated to hypertension, and this association is more often observed in men than in women. These findings suggest that high levels of both uric acid and plasma XOR activity in men play a prevalent role in gout and hypertension. However, hyperuricemia seems to be more dangerous for women than for men. Several studies carried out in different countries around the world indicate that hyperuricemia is associated with a higher risk of PAD in women but not in men. Moreover, the link between CVD and the level of both plasma XOR activity and uricemia is confirmed by the results of various investigations that report this correlation, in particular, in post-menopausal women, when the protection given by estrogen disappears. Considering the age of the subjects, the incidence of cardiovascular diseases appears to largely depend on the hormonal asset of the patients, suggesting a fundamental role of estrogens in the protection of endothelial function. Age seems to also be a crucial factor in the gender differences observed on the association of hyperuricemia with CKD.

In the same way, the relevance of the role of sex hormones in the occurrence of metabolic alterations is underlined by the prevalence of metabolic syndrome in men before 60 years and in women later. Hyperuricemia was significantly associated with high blood pressure, low high-density lipoprotein levels, high triglycerides, high low-density lipoprotein levels, high fasting plasma glucose, and central obesity in both men and women. Studies on both animal models and humans also indicate a major role of oxidative stress in the occurrence of diabetes and suggest that the serum level of XOR could be a better marker than uricemia in predicting the onset of diabetes. Although gender differences are reported in the association between hyperuricemia and both CKD and metabolic diseases, a unifying explanation has still not been found.

The control of uricemia using ULT is discouraged in the presence of asymptomatic hyperuricemia because of the possible negative effects of inhibiting XOR activities or lowering the uricemia too much, together with the occurrence of other severe drug side effects.

The differences shown in the gender distribution of the above-reported pathologies suggest the necessity of an accurate evaluation of the serum level of uric acid. In the presence of such pathological conditions associated with hyperuricemia, the ULT must be undertaken promptly, in particular, for women. In order to prevent these pathologies, further studies are desirable to ascertain the optimal levels of uricemia for men and women and to better orient the best moment for starting ULT. To overcome the contrasting results described above, we underline the need for thorough multivariate meta-analysis estimating the multi-parameter associations obtained from different studies, while also taking into account differences in the ethnicities, lifestyles, and diets of the studied groups.

## Figures and Tables

**Table 1:** Summarizing list of main results.

Pathology	Gender Correlation of Pathology with Serum Level of XOR Activity and/or Uric Acid
Gout	Hyperuricemia plays a prevalent role in men
Hypertension	High levels of both uric acid and XOR activity play a prevalent role in men
Vascular diseases	Hyperuricemia is associated with a higher risk in post-menopausal women than in men
Cardiac diseases	High levels of both uric acid and XOR activity are associated with a higher risk in post-menopausal women than in men
Kidney diseases	Hyperuricemia plays a role in both genders, although with countervailing results
Metabolic diseases	High levels of both uric acid and XOR activity play a role in both men and post-menopausal women

## Data Availability

No new data were created. All the data reported in the paper are collected from published scientific papers.
